# Chronic endometritis diagnosis and fertility outcomes: an old unresolved question

**DOI:** 10.1530/RAF-25-0016

**Published:** 2025-10-01

**Authors:** Johanna Ilic, Jessica Issa, Justine Varinot, Jerome Bouaziz, Nathalie Massin, Bassam Haddad, Cyril Touboul, Rana Mitri-Frangieh, Emile Daraï, Yohann Dabi

**Affiliations:** ^1^ Department of Obstetrics Gynecology and Reproductive Medicine, Centre Hospitalier Intercommunal de Créteil, Créteil, France; ^2^ Department of Anatomical Pathology, Centre hospitalier intercommunal de Créteil, Créteil, France; ^3^ Sorbonne University, Department of Anatomical Pathology, Tenon Hospital, Assistance Publique des Hôpitaux de Paris (APHP), Paris, France; ^4^ Department of Obstetrics Gynecology and Reproductive Medicine, Medical Center PointGyn – OneClinic, Plaisir, France; ^5^ Department of Obstetrics and Gynecology, Hôpital Tenon, Assistance Publique des Hôpitaux de Paris (APHP), Paris, France; ^6^ Sorbonne University - Department of Obstetrics and Gynecology, Tenon Hospital, Assistance Publique des Hôpitaux de Paris (APHP), Paris, France

**Keywords:** chronic endometritis, hysteroscopy, conventional histology, CD138 immunostaining, antibiotic therapy, fertility

## Abstract

**Abstract:**

Chronic endometritis, defined by chronic inflammation of the endometrium, remains a clinical and biologic challenge even using hysteroscopy allowing a direct vision of the uterine cavity without anesthesia, and conventional histology using Hematoxylin and Eosin staining. Our primary objectives were to evaluate the relevance of hysteroscopy and conventional histology compared to immunohistochemical expression of syndecan-1 (CD138, a marker of plasma cells), which is a heparan sulfate proteoglycan involved in inflammation and enables diagnosis of chronic endometritis. The second objective was to evaluate the impact of antibiotics on pregnancy rate. A retrospective study was conducted involving infertile women undergoing hysteroscopy and endometrial biopsy. Chronic endometritis was assessed using hysteroscopic findings and conventional histology compared to CD138 immunostaining. Effects of antibiotic therapy on CD138 expression on a second biopsy and on pregnancy rate were evaluated. Among the 661 infertile patients, 51 underwent hysteroscopy and endometrial biopsy. Twenty-three had a normal uterine cavity (45%) and among 28 patients with abnormal uterine cavity, ten (35.7%) had hysteroscopic findings of chronic endometritis. Sensitivity, specificity, positive predictive value, negative predictive value, and accuracy of the hysteroscopy were 22, 100, 100, and 17%, with an infinite OR and an accuracy of 68.6, and 61.4, 100, 100%, 2.9%, and 66.7% respectively for conventional histology. The correlation coefficient between the first and second reading following CD138 immunostaining was moderate (Cohen’s Kappa: 0.44 (95% CI: −0.059; 0.767) but good for plasma cell quantification (intraclass correlation coefficient 0.948). Plasma cell count was not predictive of pregnancy rate (*P* = 0.65) with an OR of 1.00. Pregnancy rate was significantly higher in treated patients (53%, 10/19) than in untreated patients (20%, 5/25) with an OR of 4.4 (95% CI: 1.17–16.8; *P* = 0.03).

**Summary:**

Chronic endometritis is a reversible cause of infertility and remains a clinical and biologic challenge even using hysteroscopy and conventional histology and relies on the presence of plasma cells in immunohistochemistry. Our results underline the low accuracy of hysteroscopy and conventional histology to assess chronic endometritis, thus supporting the systematic use of CD138 immunostaining in infertile women even in the case of normal endometrium. Moreover, pregnancy rate seems enhanced by antibiotic therapy.

## Introduction

The concept of chronic endometritis (CE) was introduced at the beginning of the 20th century by Hitschmann and Adler, evaluating the impact of inflammation on morphological endometrial features throughout the menstrual cycle (Frobenius 1990). CE is defined by a localized infectious/inflammatory disorder of the uterine mucosal lining (Greenwood & Moran 1981, Crum *et al.* 1983).

The prevalence of CE is estimated at 3–72% according to series, raising the issue of the accuracy of diagnostic criteria (Vasudeva *et al.* 1972, Crum *et al.* 1983, Han *et al.* 2024). Clinically, the most common symptoms of CE are metrorrhagia, followed by pelvic pain and leukorrhea (Kitaya & Yasuo 2011). However, most patients are asymptomatic and diagnosed in the setting of infertility (Bouet *et al.* 2016, Park *et al.* 2016). Indeed, CE is thought to be involved in infertile women (Cicinelli *et al.* 2005*a*,*b*, Kasius *et al.* 2011, Kitaya *et al.* 2012), recurrent miscarriage (Johnston-MacAnanny *et al.* 2010, Kitaya & Yasuo 2011, Zolghadri *et al.* 2011, Cicinelli *et al.* 2014, McQueen *et al.* 2015), and repeated implantation failure (Johnston-MacAnanny *et al.* 2010, Bouet *et al.* 2016, Kitaya *et al.* 2017) in 2.8%–56.8%, 9.3–67.6%, and 7.7–67.5%, respectively.

CE diagnosis remains a major diagnostic challenge. Among the various tools to assess CE, hysteroscopy, allowing a direct vision of the uterine cavity, is often recommended, showing hyperemia, a ‘strawberry’ appearance suggestive of extensive hyperemic endometrium with localized central white spots scattered throughout the cavity, stromal edema, and micropolyps (Tsonis *et al.* 2021). Among hysteroscopic features suggestive of CE, a debate exists on the most relevant (Furui *et al.* 2024). Micropolyps (less than 1 mm), often associated with stromal edema, endometrial thickening, and periglandular hyperemia, seem the most suggestive of CE (Cicinelli *et al.* 2005*a*,*b*, Peng *et al.* 2022). However, a normal hysteroscopy cannot rule out CE diagnosis (Vitagliano *et al.* 2021). Moreover, a high discrepancy rate in CE diagnosis was found between hysteroscopy and conventional histology using Hematoxylin and Eosin staining (Guo *et al.* 2021). Finally, a high intra- and inter-observer variability in hysteroscopy CE diagnosis was reported, reinforcing the need for additional consensual diagnostic criteria (Kitaya & Yasuo 2011).

Endometrial sampling with conventional histology has been used to assess CE based on the presence of endometrial stroma infiltration by plasma cells, spindled cell alteration, lymphoid follicles, stromal breakdown, or disturbance in normal endometrial growth and maturation (Vanderstraeten *et al.* 2015, Cicinelli *et al.* 2017). Using Hematoxylin and Eosin (HE) staining, plasma cells are characterized by the presence of clockwise chromatin within an eccentric nucleus with a perinuclear halo (Alonso *et al.* 2020). Immunohistochemistry (IHC) has been suggested to improve CE diagnosis by identifying plasma cells exhibiting expression of syndecan-1 (CD138, a marker of the plasma cell membrane), which is a heparan sulfate proteoglycan involved in inflammation (Bayer-Garner & Korourian 2001, Bayer-Garner *et al.* 2004).

However, a debate exists on the most adequate measurement method and on threshold determination, especially in the context of infertile women (Bayer-Garner *et al.* 2004, Bouet *et al.* 2016, Li *et al.* 2021). Subsequently, McQueen *et al.* proposed defining CE by the presence of one or more plasma cells per ten high-power fields (HPFs) (McQueen *et al.* 2021).

Therefore, using CD138 positivity as the gold standard of CE, the objectives of the present study were to correlate hysteroscopic and conventional histologic findings with CD138 values for the diagnosis of CE, to evaluate interobserver agreement, and the relation between CD138 expression and fertility outcomes.

## Materials and methods

### Population

From January 2018 to August 2020, we conducted a retrospective study from a prospective database at the Centre Hospitalier Intercommunal de Créteil (CHIC), France, to evaluate the diagnosis and impact of CE on pregnancy outcomes in infertile women. The epidemiologic characteristics of the patients were recorded.

Inclusion criteria were individuals aged 18–45 years, treated for primary or secondary infertility, who underwent both hysteroscopy and endometrial biopsy, and who were covered by French health insurance and fluent in spoken and written French.

Exclusion criteria included recent use (within 2 months before biopsy) of antibiotics or anti-inflammatory medications, need for oocyte or sperm donation, undergoing hysteroscopy without endometrial biopsy or vice versa, a delay of more than 1 month between biopsy and hysteroscopy, and clinical signs suggestive of CE.

Clinical data were abstracted from patient charts, including epidemiologic and socio-demographic factors, type of infertility (primary or secondary), smoking, gynecologic symptoms, hysteroscopic findings, and treatment received for CE.

In our department, eight gynecology specialists in assisted reproductive techniques (ART) performed hysteroscopies. All physicians had more than 3 years of experience in hysteroscopy. Approval for the study was obtained from the ethical committee of the CNGOF (Collège National des Gynécologues et Obstétriciens Francais) (CEROG: 2022-GYN-0307) and all patients gave their consent for the study.

### Methods

#### Histology protocol

On conventional histology, the CE diagnosis is based on the presence of endometrial stromal plasmacytes (ESPCs), typically appearing as large lymphocytes with a high nucleus/cytoplasm ratio, basophilic cytoplasm, and eccentric nuclei with heterochromatin rearrangement called the ‘spoke-wheel’ or ‘clock-face’ pattern. However, other endometrium-component cell types, such as NK cells, macrophages, and stromal fibroblasts, may exhibit a morphological appearance of ESPCs (Kitaya *et al.* 2018). In this specific setting, syndecan-1 (also known as CD138), a heparan sulfate/chondroitin sulfate proteoglycan expressed on the plasma membrane of ESPCs, is especially relevant to assess CE diagnosis. Using immunohistochemistry (IHC) for CD138 (IHC-CD138) markedly (odds ratio: 2.8) improves the sensitivity (100 vs 75%), specificity (100 vs 65%), interobserver variability (96 vs 68%), and intra-observer variability (93 vs 47%) in the histologic diagnosis of CE, supporting its use as the most accurate diagnostic tool (Miguel *et al.* 2011, Kitaya & Yasuo 2013). As such, the CD138 immunostaining was considered the gold standard for CE diagnosis.

Concerning the ESPC cut-off to diagnose CE, no clear consensus exists. Despite some limits, in accordance with previous studies (Hirata *et al.* 2021, McQueen *et al.* 2021), we decided to use the cut-off of ESPC ≥1 in ten HPFs.

Histology was performed on endometrial biopsy using a Cornier device during a clinical visit or at the end of the hysteroscopy.

For conventional histology examination, specimens were stained by HE. Briefly, for IHC, 4 μm slides were obtained from formalin-fixed paraffin-embedded (FFPE) tissues and were deparaffinized and dehydrated using alcohol.

IHC staining was performed using an automated system (Ventana, BenchMark ULTRA, 315485, Roche, Switzerland), and a dilution of 1:250 was used for the mouse anti-CD138 antibodies (760-4248 Roche) in accordance with laboratory recommendations. The immunostaining was carried out with 3, 3′-diaminobenzidine chromogen (DAB) and counterstained with hematoxylin. The sections were then incubated with a 1:250 dilution of mouse anti-CD138 antibody (Gene Tech, clone B-A38, China). Subsequently, the slides were washed and incubated with a horseradish peroxidase-conjugated secondary antibody for 30 min.

The slides were read under the microscope at ×40 magnification, and the plasma cell count was performed with the CaloPix software (CaloPix 4.1.0.9, TRIBVN, France). Two independent pathologists read the slides and performed plasma cell counts. In case of discrepancy between pathologists in either CE diagnosis or plasma cell count, a third pathologist reviewed the slides. In accordance with a previous study, the diagnosis of CE was considered positive when at least one plasma cell was observed on each slide at ten HPF (McQueen *et al.* 2021).

#### Hysteroscopic criterion of CE

The material used was rigid hysteroscopy STORZ, 30°, 4.1 mm, GmbH Germany.

In 2019, based on a systematic review and the agreement of the Delphi poll, the International Working Group for the Standardization of CE Diagnosis proposed the following hysteroscopic diagnostic criteria for CE (Cicinelli *et al.* 2019):

- Strawberry aspect: first described by Cravello *et al.* (1997), recognized as large hyperemic localized or scattered mucosal areas flushed with white central points.
- Focal hyperemia: small areas of hyperemic mucosa.
- Hemorrhagic spots: focal reddish mucosa with sharp and irregular borders, possibly in continuity with capillaries.
- Endometrial micropolyps: first described by Cicinelli *et al.* (2005*a*,*b*), typically visualized as a cluster of less than 1 mm-sized protrusions on the focal or entire mucosal surface with a distinct connective vascular axis.
- Stromal edema: thick and pale appearance of the mucosa in the follicular phase originating from the stromal compartments (a normal finding during the secretory phase).

#### Antibiotic protocol

Antibiotic treatment with Roxithromycin for 10–15 days was administered. None of the patients had a pro/prebiotic treatment.

No microbiology assessment was performed on the study population.

#### Statistical analysis

The data were collected on an Excel worksheet (Microsoft Corporation, USA), and all statistical analyses were performed using R studio software (version 1.3.1093, freely available online).

Comparison for qualitative variables was assessed by the Chi-square test or Fisher’s exact test and by the Student’s *t*-test for quantitative variables. A *P*-value <0.05 was considered to denote a significant difference.

Sensitivity is the ability of a test to correctly identify patients with a disease. Specificity is the ability of a test to correctly identify people without the disease. True positive is when the person has the disease and the test is positive. True negative is when the person does not have the disease and the test is negative.

Inter-observer agreement analysis was performed using Cohen’s quadratic Kappa test for the diagnosis of CE and the intraclass correlation coefficient for the correlation of plasma cell count. This agreement is considered excellent when Kappa is between 1 and 0.8, good between 0.61 and 0.80, average between 0.41 and 0.6, poor between 0.21 and 0.40, and bad between 0 and 0.20.

## Results

### Study population

**Figure 1:**
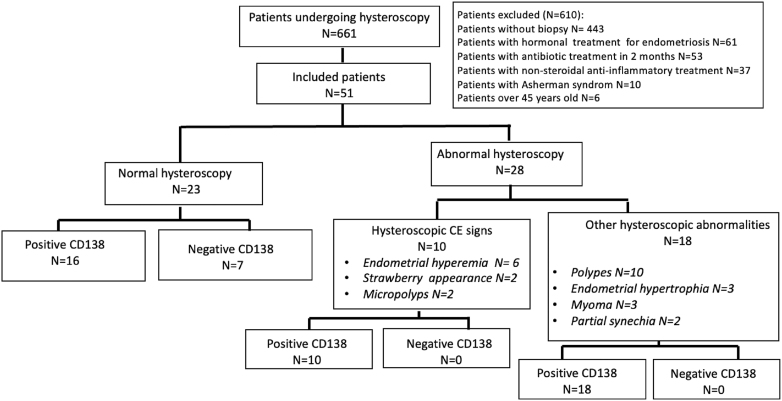
Flow chart of the study.

During the inclusion period, 661 infertile patients underwent a hysteroscopy. Among them, 610 were excluded due to a normal appearance of the uterine cavity without systematic endometrial biopsy (*n* = 443). The other exclusion criteria were women with hormonal treatment for endometriosis (*n* = 61), women with antibiotic therapy for non-gynecologic reasons during the 2 months before hysteroscopy (*n* = 53), women with long-term NSAID treatment (*n* = 37), women with severe Asherman syndrome (*n* = 10), and women over 45 years old (*n* = 6). Therefore, the study population was composed of 51 women (7.7%) who underwent both a hysteroscopy and an endometrial biopsy (Fig. 1). The epidemiologic characteristics of the study population are summarized in Table 1. Median age was 36 years (range: 26–44 years). Concomitant diagnosis of deep infiltrating endometriosis was noted in 12% of patients.

**Table 1:** Characteristics of the overall study population and according to CE status. Data are presented as *n* (%) or as median (min–max).

	Whole population	Patients with CE	Patients without CE	*P* value
Total *n*	51	44	7	
Age, years	35.2 (26–44)	35.5 (26–44)	33.5 (29–38)	0.16
BMI, Kg/m^2^	26.3 (17–40)	26.8 (17–40)	23.1 (18–32)	0.15
Smoking	10 (20)	8 (18)	2 (29)	0.61
Endometriosis	6 (12)	5 (11)	1 (14)	1.0
Nulliparous	37 (73)	30 (68)	7 (100)	0.16
Prior miscarriage	23 (45)	19 (43)	4 (57)	0.68
Primary infertility	20 (39)	18 (41)	2 (29)	0.69
Secondary infertility	31 (61)	26 (59)	5 (71)	0.69
Infertility duration (months)	73.8 (7–780)	71.3 (7–780)	89.1 (24–168)	0.36
ART failure	8 (16)	7 (16)	1 (14)	1.0

BMI, body mass index; ART, assisted reproductive technology.

### CD138 analysis

Using CD138 immunostaining, 44 (86.2%) patients had a CE diagnosis and seven had no CE based on negative CD138 expression. The mean number of plasma cells per HPF was 155.9. The distribution of CD138 immunostaining was less than 5, between 5 and 50, and over 50 in 12 (27.2%), 19 (43.1%), and 13 (29.5%) patients, respectively. The correlation coefficient between the first and second reading for the diagnosis of CE (presence or absence of plasma cells) was moderate (Cohen’s Kappa: 0.44 (95% CI: −0.059; 0.767)).

A discrepancy in plasma cell counts by the two pathologists of <5% was observed in 23.5% of cases, between 5 and 20% in 15.7%, and >20% in 54.9% of cases.

Therefore, the correlation coefficient between the first and second reading for plasma cell quantification was good (intraclass correlation coefficient: 0.948).

### Relevance of hysteroscopic and conventional histologic findings to assess CE

Among the 51 patients of the study population, 23 had a normal uterine cavity appearance (45%). For the 28 patients with abnormal uterine cavity, ten (35.7%) had hysteroscopic findings suggestive of CE, mainly corresponding to endometrial hyperemia areas in six patients, strawberry appearance in two patients, and micropolyps in only two patients (Fig. 1). The sensitivity, specificity, PPV, NPV, and accuracy of hysteroscopy to diagnose CE were 22, 100, 100, and 17%, with an infinite OR and an accuracy of 68.6%, respectively.

Conventional histologic analysis was blinded to hysteroscopic findings. At conventional histology, the average slide area analyzed was 152 mm^2^ (range: 10–427). Using HE, histology allowed to diagnosis of CE in 54% (28/51) of patients.

The sensitivity, specificity, PPV, NPV, and the accuracy of conventional histology were 61.4, 100, 100, 29, and 66.7%, respectively.

### Diagnostic value of hysteroscopic findings and conventional histology to diagnose CE compared to CD138 expression

Of the 23 patients with normal uterine cavity appearance, 16 (69.5%) had positive CD138 immunostaining. For the ten patients with hysteroscopic findings suggestive of CE, all had positive CD138 immunostaining. For the remaining 18 (64.2%) patients with uterine cavity abnormality (polyp, myoma, partial synechia, and endometrial hypertrophy) but without hysteroscopic CE signs, as defined in the material and methods, all had positive CD138 immunostaining.

### Comparison of epidemiologic characteristics between women with and without CE

The epidemiologic characteristics of the patients according to the CE diagnosis based on CD138 positivity are summarized in Table 1. No differences were observed between the groups, except for a higher BMI, a lower nulliparous rate, and a shorter duration of secondary infertility observed in the CE group (Table 1).

### Changes in plasma cell count after antibiotic therapy

Among the 44 patients with CE, 19 (43%) were treated with antibiotics. The indication for antibiotic treatment was left to the discretion of the ART physician.

During follow–up, 27.2% (12/44) underwent a second biopsy, including five patients treated with antibiotics and seven untreated. On the second biopsy in untreated patients, three of seven patients had an increased plasma cell count, while a spontaneous decrease or negative plasma cell count was noted in the remaining four patients (Annex 1 (see section on Supplementary materials given at the end of the article)). On the second biopsy of treated patients, one had a negative plasma cell count and four had a decreased plasma cell count. Median interval between the two biopsies was 12 months (range: 4–17 months) (Annex 1).

### Relation between antibiotic treatment, plasma cell counts, and pregnancy rate (PR)

Among patients with CE, 34% (15/44) achieved pregnancy. The PR was significantly higher in patients treated with antibiotic (53%, 10/19) than in untreated patients (20%, 5/25) with an OR of 4.4 (95% CI: 1.17–16.8; *P* = 0.03). The median time to achieve pregnancy was 12 months (range: 11–36 months).

The plasma cell count at diagnosis was not predictive of achieving pregnancy (*P* = 0.65) with an OR of 1.00. Among pregnant patients, the plasma cell count was less than 50 in 80% of patients (12/15).

## Discussion

The present study has demonstrated the low relevance of hysteroscopy to suggest CE, even in the presence of micropolyps. Moreover, conventional histology had low accuracy to assess the CE diagnosis. Antibiotic therapy confirms its relevance to enhance PR.

CE diagnosis is a major challenge for infertile women due to the risk of increased implantation failure (Kushnir *et al.* 2016, Cicinelli *et al.* 2018). In the present study, including infertile women without symptoms of uterine infection, the rate of CE on hysteroscopy was only 19.6% (10/51). Moreover, hysteroscopy was considered normal in 36% of patients with CE, supporting systematic CD138 staining in the context of infertility. In the patients with hysteroscopic signs suggestive of CE, hyperemia was the most relevant feature, while the presence of micropolyps was observed in only 20% cases, raising several issues on CE diagnosis criteria. Indeed, micropolyps (<1 mm) diagnosis is easy in case of multi-micropolyps, but difficult in case of isolated micropolyps. Moreover, as previously suggested, the experience of physicians performing hysteroscopy can be discussed (Cicinelli *et al.* 2019). In our series, all hysteroscopies were performed by physicians with high experience. Our results contrast with those of Cicinelli *et al.* (2005*a*,*b*) showing the relation between micropolyps and CE. These authors reported an increased likelihood of CE in women with micropolyps with an OR of 124.2 (95% CI: 50.3–205.4). Their respective sensitivity, specificity, positive and negative predictive values of micropolyps to predict CE were 54, 99, 94, and 89%, and a diagnostic accuracy of 90%. However, our results agree with those of Furui *et al.*, showing that endometrial congestion was the only hysteroscopic finding significantly associated with CE (Furui *et al.* 2024). To overcome the diagnostic gap between hysteroscopy findings and histology to assess CE, using logistic regression analysis, Song *et al.* showed that hyperemia area degree ≥2, micropolyps, polypoid hyperplasia, and history of ectopic pregnancy were independent risk factors for CE, allowing development of a nomogram with a ROC curve of 0.801 (95% CI: 0.742–0.861) (Song *et al.* 2019). Interestingly, Kitaya *et al.* developed a deep learning model (ARChival) of hysteroscopic image-based prediction for CE in infertile women, exhibiting a respective accuracy, AUC, sensitivity, and specificity of 0.845, 0.98, 77.97%, and 100%, but requiring external validation (Kitaya *et al.* 2024). Finally, another crucial data of the present study is the association between CE and the presence of uterine cavity abnormality, underlining the need to manage these women with systematic antibiotic therapy.

Recent guidelines on indications and techniques for endometrial biopsy to diagnose CE have recommended the use of a grasp biopsy technique as the first choice in reproductive-aged women (Vitale *et al.* 2023). However, no clear histologic criteria have been suggested to affirm CE diagnosis. In the present study, low relevance of conventional histology was observed to diagnose CE, with a sensitivity, specificity, PPV, NPV, and accuracy of 61.4, 100, 100, 29, and 66.7%, respectively. This can be linked to difficulties in identifying plasma cells by conventional histology, which is considered the gold standard for CE diagnosis (Chen *et al.* 2016, Li *et al.* 2021). Our results are in line with those of Pérez-Cejuela *et al.*, showing a low agreement between hysteroscopic findings and conventional histology, with a Cohen’s kappa index of 34% (Pérez-Cejuela *et al.* 2023). These results underline the usefulness of combining hysteroscopy and endometrial sampling with IHC to assess CE. In the current study, using CD138 immunostaining, a large range of plasma cells from 0 to over 1,000 was observed, with perfect concordance in plasma cell counts according to readers in only one-third of cases. When CE diagnosis was based on the presence of at least one plasma cell, the correlation coefficient between readers was moderate (Cohen’s Kappa: 0.44 (95% CI: −0.059; 0.767)), underlying the need for additional tools such as the contribution of deep learning artificial intelligence analysis (Greeley *et al.* 2024). However, the correlation coefficient between the first and second reading for plasma cell quantification was good (intraclass correlation coefficient: 0.948). In this specific setting, previous studies have underlined the contribution of multiple myeloma antigen 1 (MUM-1) evaluation, which is expressed on plasma cells, activated B cells, and T cells (Falini *et al.* 2000, Wasco *et al.* 2008).

Another concern is how to evaluate the impact of antibiotic therapy on PR. In the present study, despite a small sample size, PR was higher in patients treated with antibiotics vs untreated patients, with an OR of 4.4 (95% CI: 1.17–16.8; *P* = 0.03). Our results agree with those of a retrospective study showing the relevance of evaluating and treating CE before IVF procedure on PR (Vaduva *et al.* 2023). Moreover, the randomized trial of Cicinelli *et al.* demonstrated the positive impact of antibiotic therapy vs no treatment for CE on PR (Cicinelli *et al.* 2021). Similarly, Dang *et al.* showed that the PR in the CD138-positive group was lower compared to the CD138-negative group (64.79 vs 81.30%, *P* < 0.05) (Dang *et al.* 2024). Using multivariate analysis, these authors demonstrated that CD138 positivity was an independent risk factor to predict embryo implantation failure. However, no evaluation of CD138 changes after antibiotics was performed. In the current study, 12 patients underwent a second endometrial biopsy: five patients after antibiotic treatment and seven untreated. Among patients treated, only one of five had negative CD138 immunostaining, and the remaining four patients had a decrease, while in untreated patients, more than half (four of seven) had spontaneous decrease or negative plasma cells. However, our results of the second biopsy should be analyzed with caution due to the large range of delay between antibiotic treatment and the second biopsy. These data raise issues on how to evaluate the efficacy of antibiotics on CE and factors contributing to spontaneous CD138 immunostaining decrease in untreated patients. A potential explanation is linked to desquamation of endometrium during menstruation (Lozano *et al.* 2021). Faced with this issue, Lozano *et al.* evaluated vaginal and endometrial microbiota in patients with and without CE (assessed by CD138 immunostaining), revealing an abundance of Ralstonia and Gardnerella in endometrial samples, and of Streptococcus and Ureaplasma in vaginal samples (Lozano *et al.* 2021). Moreover, Han *et al.* identified four microbial vaginal markers of CE (Enterobacter, Prevotella, Faecalibacterium, and Phascolarctobacterium), allowing development of a predictive classifier for CE diagnosis with an AUC of 83.26% (Han *et al.* 2024). When focusing on infertile women, Tanaka *et al.* suggested that vaginal microbiota of patients with CE exhibited a reduction in *Bifidobacterium* and of lactic-acid, not *Lactobacillus*-dependent, allowing identification of patients with potential benefit from antibiotic treatment (Tanaka *et al.* 2022).

Some limits of the present study deserve to be underlined. First, the retrospective nature cannot exclude all potential biases. Second, the small sample size limits drawing definitive conclusions on systematic hysteroscopy compared to endometrial biopsy alone to assess CE diagnosis. Third, a high variation in the delay to perform a second biopsy was observed due to the absence of a strict protocol. This could be a bias related to potential CE recurrence. Fourth, no attempt was made to evaluate the contribution of MUM-1 immunostaining to improve CE diagnosis. However, previous studies have demonstrated that the plasmacyte marker CD138 is currently the most reliable and time-saving diagnostic method for CE (Kannar *et al.* 2012, Kitaya *et al.* 2012). Finally, further studies are required to identify markers of CE response to antibiotics. In this specific setting, Di Pietro *et al.* reported that upregulation of miRNA-27a-3p and miRNA-124-3p in endometrium and serum from patients with CE could be new potential molecular markers (Di Pietro *et al.* 2018). Moreover, Wang *et al.* suggested that exosomes derived from adipose tissue-derived stem cells could exert an anti-inflammatory effect on endometrial cells via the miR-21/TLR4/NF-kB signaling pathway (Wang *et al.* 2023).

## Conclusion

Despite some limits of the present study, our results underlined the low relevance of hysteroscopic features, including micropolyps, to suggest CE. However, even in case of normal endometrial appearance, infertile patients might benefit from a systematic evaluation of CE, as antibiotic therapy seems to have a positive impact on the PR. Further studies should clarify the role of antibiotics in improving fertility outcomes in this specific setting.

## Supplementary materials



## Declaration of interest

The authors declare that there is no conflict of interest that could be perceived as prejudicing the impartiality of the work reported.

## Funding

This research did not receive any specific grant from funding agencies in the public, commercial, or not-for-profit sectors.

## Author contribution statement

JI and YD had the original idea for the study and contributed to study design. JI, JI, JV, JB, and NM collected data. JI and YD performed statistical analysis. JI, YD, and ED drafted the paper. ED, CT, and BH supervised the study. All authors contributed to the interpretation of the data, revisions, and gave input at all stages of the study. All authors have read and agreed to the published version of the manuscript.
