# Supplementary material for: Chronic endometritis diagnosis and fertility outcomes: an old unresolved question
Source: Reprod Fertil. 2025 Oct 1;6(4):e250016. doi: 10.1530/RAF-25-0016 (PMC12495947; doi:10.1530/RAF-25-0016)
Supplement: Supplementary file 1 [file supplementary_materials.pdf]

		First biopsy<br>plasma cells/mm2	Second biopsy<br>plasma cells/mm2	Time (month)
**Treated patients**	Patient 1	0. 81	0.05	14
	Patient 2	0.21	0	12
	Patient 3	1.95	0.23	12
	Patient 4	38	12	4
	Patient 5	0.04	0.06	6
**Untreated patients**	Patient 1	0.05	0.08	11
	Patient 2	6.9	0	4
	Patient 3	0.10	0.05	17
	Patient 4	1.06	4.08	12
	Patient 5	0.002	0	6
	Patient 6	0.01	0.02	13
	Patient 7	0.04	0	9

Annex 1: Plasma cell count on first and second biopsy in untreated and treated patients by antibiotic therapy
